# Arbutin suppresses osteosarcoma progression via *miR‐338‐3p*/MTHFD1L and inactivation of the AKT/mTOR pathway

**DOI:** 10.1002/2211-5463.13024

**Published:** 2020-12-03

**Authors:** Cheng‐Qun Wang, Xiu‐Mei Wang, Bing‐Liang Li, Yuan‐Min Zhang, Lei Wang

**Affiliations:** ^1^ Department of Joint Surgery Affiliated Hospital of Jining Medical University China; ^2^ Electroencephalogram Room Affiliated Hospital of Jining Medical University China

**Keywords:** AKT/mTOR, Arbutin, *miR‐338‐3p*, *MTHFD1L*, osteosarcoma, proliferation

## Abstract

Arbutin, a glycoside extracted from the plant *Arctostaphylos uva‐ursi*, has been previously reported to possess antioxidant, anti‐inflammatory and anticancer effects. Here, we investigated whether arbutin affects the proliferation of the cells of the osteosarcoma (OS) cell lines MG‐63 and SW1353. Arbutin suppressed OS cell viability in a dose‐ and time‐dependent manner, as shown by Cell Counting Kit‐8 assay. Furthermore, arbutin exposure decreased the protein levels of MTHFD1L, CCND1 and phosphorylated‐protein kinase B (AKT)/phosphorylated‐mammalian target of rapamycin (mTOR). Potential upstream miRNAs of *MTHFD1L* were predicted using TargetScan, PICTAR5, miRanda and miRWalk. We performed luciferase activity assays to show that *miR‐338‐3p* directly targets and negatively regulates the expression of MTHFD1L. Knockdown of *miR‐338‐3p* promoted cell invasion, migration and proliferation in arbutin‐treated OS cells via MTHFD1L. In summary, our data suggest that arbutin inhibits OS cell proliferation, migration and invasion via *miR‐338‐3p*/MTHFD1L and by inactivating the AKT/mTOR pathway.

## Graphical abstract

Arbutin, a natural polyphenol, inhibits OS cell viability, invasion and migration by down‐regulating MTHFD1L expression. *miR‐338‐3p* directly targets MTHFD1L. This regulatory action is associated with the inactivation of the AKT/mTOR signaling pathway.

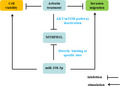

**Abbreviations**
AKT: protein kinase B
CCK‐8: Cell Counting Kit‐8
CTDbase: Comparative Toxicogenomics Database
GEO: Gene Expression Omnibus
miRNA: microRNA
mTOR: mammalian target of rapamycin
mut: mutant
NC: negative control
OS: osteosarcoma
p: phosphorylated
qRT‐PCR: quantitative real‐time PCR
SD: standard deviation
si‐: small interfering
wt: wild‐type

Osteosarcoma (OS) is a malignant neoplasm of the bone, which originates from the distal end of long bone and is most common in young adolescents [1]. Despite that prodigious progress has been achieved in OS treatment, the long‐term overall survival rate of patients with OS was still unfavorable, attributed to its sharp progression and early metastasis [2, 3]. The current main therapy is surgical resection with systemic multidrug chemotherapy, which eventually leads to serious resistance, aggravates metastasis and increases recurrence rate [4].

Increasing publications indicate that the wide utility of natural agents opens up a novel avenue for the management of cancers [5]. Arbutin, also namely as 4‐hydroxyphenyl‐β‐d‐glucopyranoside, is a natural polyphenol extracted from the bearberry plant *Arctostaphylos uva‐ursi* (molecular mass: 272 Da) [6]. It exhibits a range of biological activities. Traditionally, Arbutin is used as a skin‐whitening or depigmenting agent [7]. The antioxidant, anti‐inflammatory, and even anticancer capabilities of Arbutin have been fully focused [8]. Arbutin is able to attenuate the melanin content, as well as tyrosinase activity, in melanoma cells [9]. Previous study has indicated that Arbutin could facilitate apoptosis of human bladder cancer and melanoma A375 cells [10, 11]. Nawarak *et al*. [11] have confirmed that the repression of Arbutin to aggressive melanoma development is associated with the dysregulation of p53, ENOA and VIME. Based on our prior study, MTHFD1L has been concluded as a prospective therapeutic target for OS [12]. The Comparative Toxicogenomics Database (CTDbase) website analysis revealed that Arbutin treatment can decrease the expression of MTHFD1L. However, the effects of Arbutin in OS progression are largely unknown.

Cumulating investigations manifest that the progression of malignant OS is related to the aberrant microRNAs (miRNAs) expression. *miR‐338‐3p* serves as a tumor repressor in different human cancers, such as breast cancer [13], colorectal cancer [14], non‐small cell lung cancer [15] and prostate cancer [16]. More than that, *miR‐338‐3p* has been documented to hinder proliferation, invasion and other malignant behaviors in OS cells via targeting ATPase homolog 1 or RUNX2/CDK4 [17, 18]. Bioinformatics analysis of our work predicted that *miR‐338‐3p* may be an upstream of *MTHFD1L* in OS. It is not known, however; there are still rare investigations on its association between Arbutin and MTHFD1L in OS.

Hence we detected the pharmacological impacts of Arbutin on cell viability, invasion and migration in OS cells. Furthermore, we also evaluated whether the *miR‐338‐3p*/MTHFD1L axis is implicated in the regulatory action of Arbutin in the progression of OS.

## Materials and methods

### Arbutin and cell culture

Arbutin (cat. no HY‐N0192; purity > 98%) was obtained from MedChemExpress LLC (Monmouth Junction, NJ, USA). It was dissolved in DMSO solution (Sigma, St Louis, MO, USA) and then stored at 4 °C. Two OS cell lines (MG‐63 and SW1353) were obtained from American Type Culture Collection (Manassas, VA, USA). The culture medium was Dulbecco's modified Eagle's medium (Invitrogen, Carlsbad, CA, USA) containing 1% antibiotics (penicillin and streptomycin) and 10% FBS (Invitrogen). The culture condition was 37 °C in carbon dioxide incubator with 5% CO_2_.

### Transfection

Small interfering (si) ‐MTHFD1L to knock down MTHFD1L expression, *miR‐338‐3p* inhibitor to reduce *miR‐338‐3p* expression and their corresponding controls were all synthesized by GenePharma company (Shanghai, China). Cells (5 × 10^5^) were inoculated in six‐well plates to achieve more than 80% confluency proceeding to transfection using Lipofectamine 2000 (Life Technologies, Grand Island, NE, USA). After 24 h of transfection, quantitative real‐time PCR was conducted to quantify the *MTHFD1L* or *miR‐338‐3p* expression.

### Cell viability analysis

Cell Counting Kit‐8 (CCK‐8) (Beyotime, Beijing, China) was used to estimate the proliferative ability of cells. Cells (1000 cells for each well) treated by Arbutin were seeded in 96‐well plates in 37 °C with 5% CO_2_, followed by the treatment of CCK‐8 solution (10 μL per well) at indicated time points and 1.5‐h additional culture. The absorbance (*A*) value was recorded by a Tecan Group microplate reader (Shanghai, China) with the wavelength of 450 nm.

### Cell migration and invasion assay

Transwell chamber (8‐μm pore size; BD Biosciences, Franklin Lakes, NJ, USA) was applied to detect the migratory and invasive capabilities of OS cells. In brief, Matrigel was bio‐coated on the top surface of transwell chambers for cell invasion analysis. The treated cells were subsequently cultivated at 37 °C with 5% CO_2_ for 48 h. These cells were resuspended into Dulbecco's modified Eagle's medium without serum, 100 μL (1 × 10^5^) of cell suspension was placed into the top surface, while 500 μL of conventional culture medium with 10% FBS was put on the lower chamber. Overnight, invaded cells passed through Matrigel or migrated cells passed through polyethylene membrane were subsequently fixed by 4% paraformaldehyde. The crystal violet staining was used to show the invasive and migratory cells using a light microscope (Motic, Xiamen, China).

### Luciferase activity assay

Dual‐luciferase Reporter Assay Kit was used to determine the relevance between *miR‐338‐3p* and *MTHFD1L* in OS cells. First, the sequences of *MTHFD1L* carrying the binding sites to miR‐338‐3p or not were amplified into luciferase reporter vector pmirGLO, which was named wild‐type (wt)‐*MTHFD1L* and mutant (mut)‐*MTHFD1L*. After 24 h, cells transfected with *miR‐338‐3p* inhibitor, the specific negative control (NC) and wt‐*MTHFD1L* or mut‐*MTHFD1L* were collected to measure the luciferase activity.

### Quantitative real‐time PCR

The expression of *MTHFD1L* of transfected OS cells was examined with quantitative real‐time PCR test. Total RNA of cells with various treatments was isolated by TRIzol reagent and then reverse transcribed into cDNA by TaqMan Reverse Transcription Kit (Thermo Fisher Scientific, San Jose, CA, USA). SYBR Premix Ex Taq II (Takara, Dalian, China) was used to conduct quantitative real‐time PCR on the basis of protocols provided by the manufacturer on the 7900HT real‐time PCR system. Glyceraldehyde‐3 phosphate dehydrogenase was used as a housekeeping gene, and the relative expression of *MTHFD1L* was normalized to glyceraldehyde‐3 phosphate dehydrogenase, calculated by the $2^{‐\Delta \Delta C_{t}}$ method.

### Western blot analysis

Cell lysates were isolated with radioimmunoprecipitation assay buffer supplemented with protease inhibitor. The bicinchoninic acid method was used to detect the concentration of proteins. Afterward, proteins (20 μg) were loaded in 12% SDS/PAGE by electrophoresis and transferred onto poly(vinylidene difluoride) membranes. Followed by blocking using 5% fat‐free milk, these poly(vinylidene difluoride) membranes were first hybridized with indicated primary antibodies against MTHFD1L, CCND1, AKT/phosphorylated (p)‐AKT and mTOR/p‐mTOR overnight at 4 °C and probed with secondary antibody for 1 h at 37 °C. The protein bands were visualized with ECL Plus (Thermo Scientific, Rockford, IL, USA) and scanned by imagej software (National Institutes of Health, Bethesda, MD, USA). Glyceraldehyde‐3 phosphate dehydrogenase was selected as the loading control.

### Bioinformatics and statistics

OS clinical data from the Gene Expression Omnibus (GEO) database were downloaded for computing the differentially expressed miRNAs. TargetScan, PICTAR5, miRanda and miRWalk were used to predict the possible upstream regulators of MTHFD1L. The potential drugs that could inhibit MTHFD1L expression were achieved via accessing the CTDbase. All data were statistically analyzed by spss version 22.0 (SPSS, Chicago, IL, USA) and exhibited as mean ± standard deviation (SD). Student’s *t*‐test or ANOVA with Dunn’s test was used for comparisons in two or more groups. A *P* value <0.05 was regarded as statistically significant.

## Results

### Arbutin inhibits cell proliferation in OS cells

**Fig 1:**
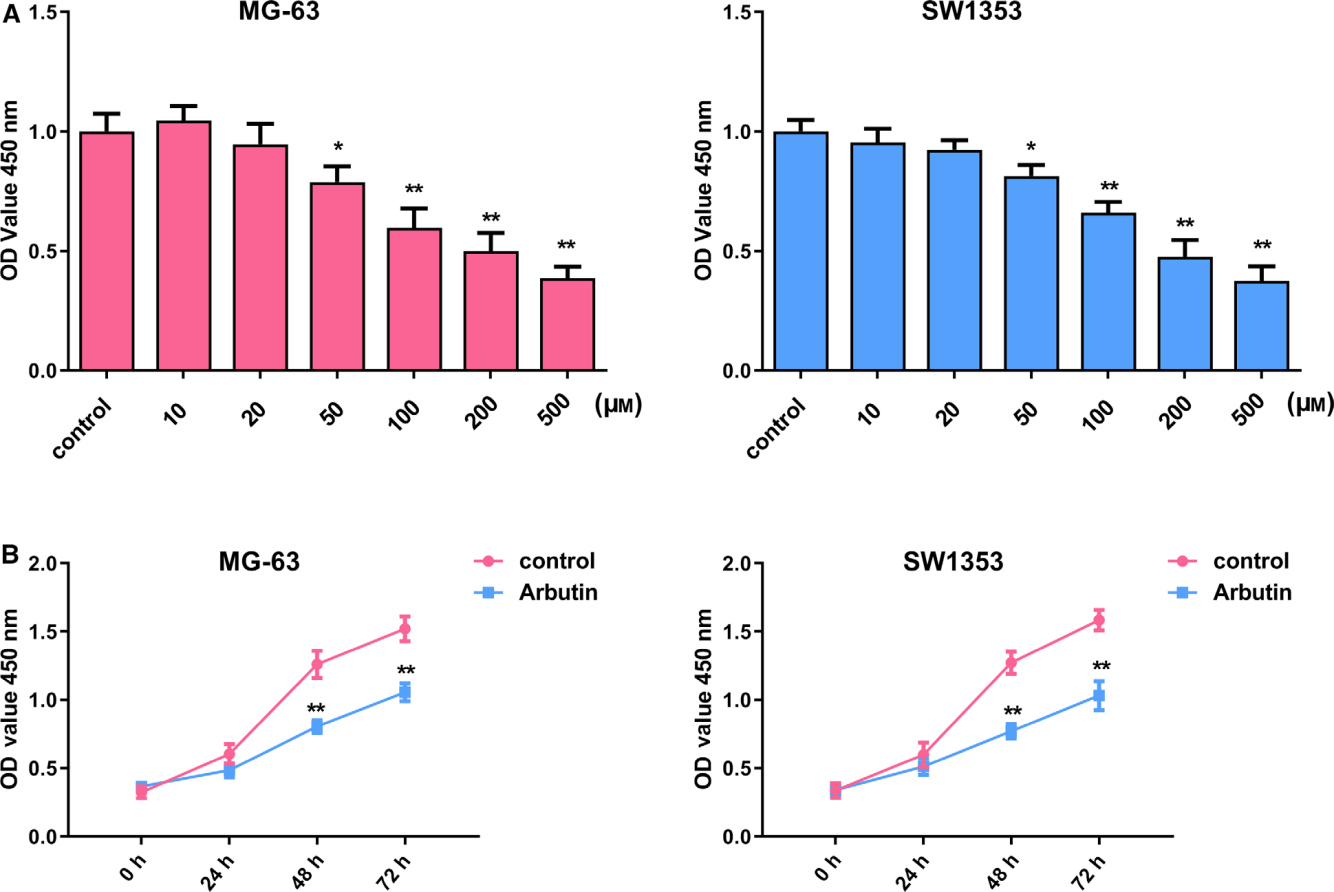
Arbutin suppresses OS cell viability. (A) The proliferative ability was explored using CCK‐8 kit in MG‐63 and SW1353 cells treated with 10, 20, 50, 100, 200 and 500 μm Arbutin. (B) The proliferation of OS cells induced by 200 μm Arbutin was measured in a time‐dependent manner by CCK‐8 assay. Data are shown as the means ± SD of three independent experiments. The data were statistically analyzed by Student’s *t*‐test. *P < 0.05, **P < 0.01 versus control group.

MG‐63 and SW1353 OS cell lines were selected to evaluate whether Arbutin affects the proliferative ability of OS cells in this study. MG‐63 and SW1353 cells were first treated by specific concentrations (10, 20, 50, 100, 200 and 500 μm) of Arbutin, and cell proliferation was examined using CCK‐8. Results manifested that the proliferative capability of MG‐63 and SW1353 cells was gradually reduced with the increasing concentration of Arbutin (Fig. 1A). The concentration of 200 μm was finally selected for further experiments. CCK‐8 assay at a time‐dependent mode showed that Arbutin (200 μm) significantly inhibited cell viability of OS cells in both MG‐63 and SW1353 (Fig. 1B). Therefore, these findings demonstrated that *in vitro* treatment of OS with Arbutin elicited significant inhibition against cell viability.

### Arbutin treatment attenuates MTHFD1L expression and might inactivate the AKT/mTOR pathway

**Fig 2:**
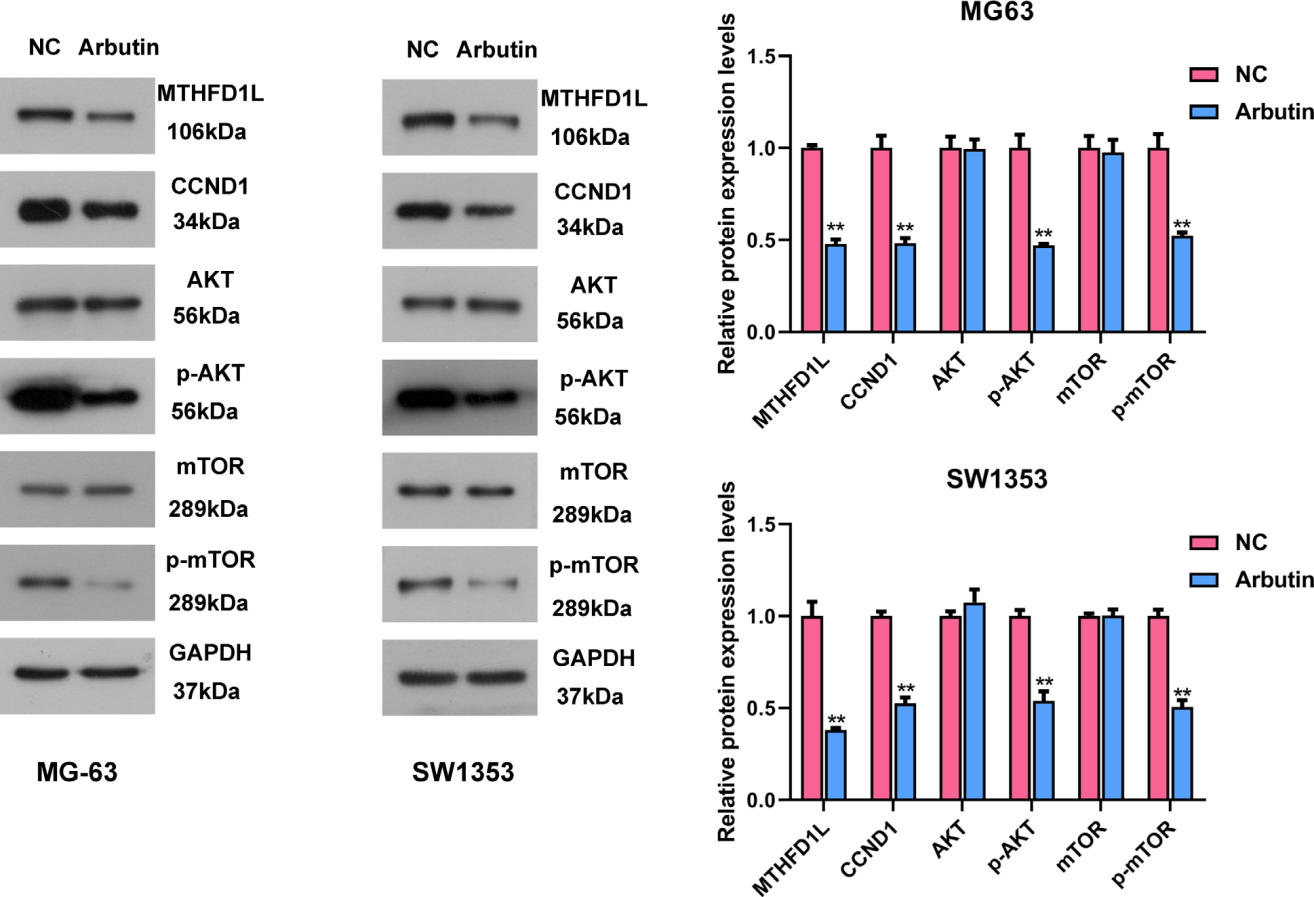
Arbutin attenuates MTHFD1L and CCND1 expression and might inactivate the AKT/mTOR pathway. The expressions of MTHFD1L, CCND1, AKT/p‐AKT and mTOR/p‐mTOR were investigated by using western blot in Arbutin‐treated OS cells. The intensity of protein bands was quantified. Data are shown as the means ± SD of three independent experiments. The data were statistically analyzed by Student’s *t*‐test. **P < 0.01 versus NC group.

Analysis of the CTDbase revealed that Arbutin was a potential agent inhibiting the expression of MTHFD1L. Thus, to verify the correlation, we implemented western blot to evaluate the MTHFD1L expression in OS cells treated by Arbutin. Results exhibited that the treatment of Arbutin significantly declined the MTHFD1L protein expression (Fig. 2). The expression of CCND1 was also measured by western blot experiment to estimate the impact of Arbutin on cell cycle in OS cells. Arbutin exposure induced a decrease in level of CCND1 in MG‐63 and SW1353 cells (Fig. 2). In addition, as we previously described [12], silencing MTHFD1L can suppress the activity of the AKT/mTOR pathway in OS cells. Thereby, we evaluated the AKT/mTOR pathway‐related protein levels to validate whether Arbutin treatment affects the activation of AKT/mTOR signaling. As illuminated in Fig. 2, the levels of p‐AKT and p‐mTOR were all reduced after Arbutin treatment, and AKT and mTOR showed no differences compared with the NC group. Taken together, these findings validated that the treatment of Arbutin attenuated MTHFD1L and CCND1 expression and restrained the AKT/mTOR signaling in OS.

### *MTHFD1L* is a direct target gene of *miR‐338‐3p* in OS cells

**Fig 3:**
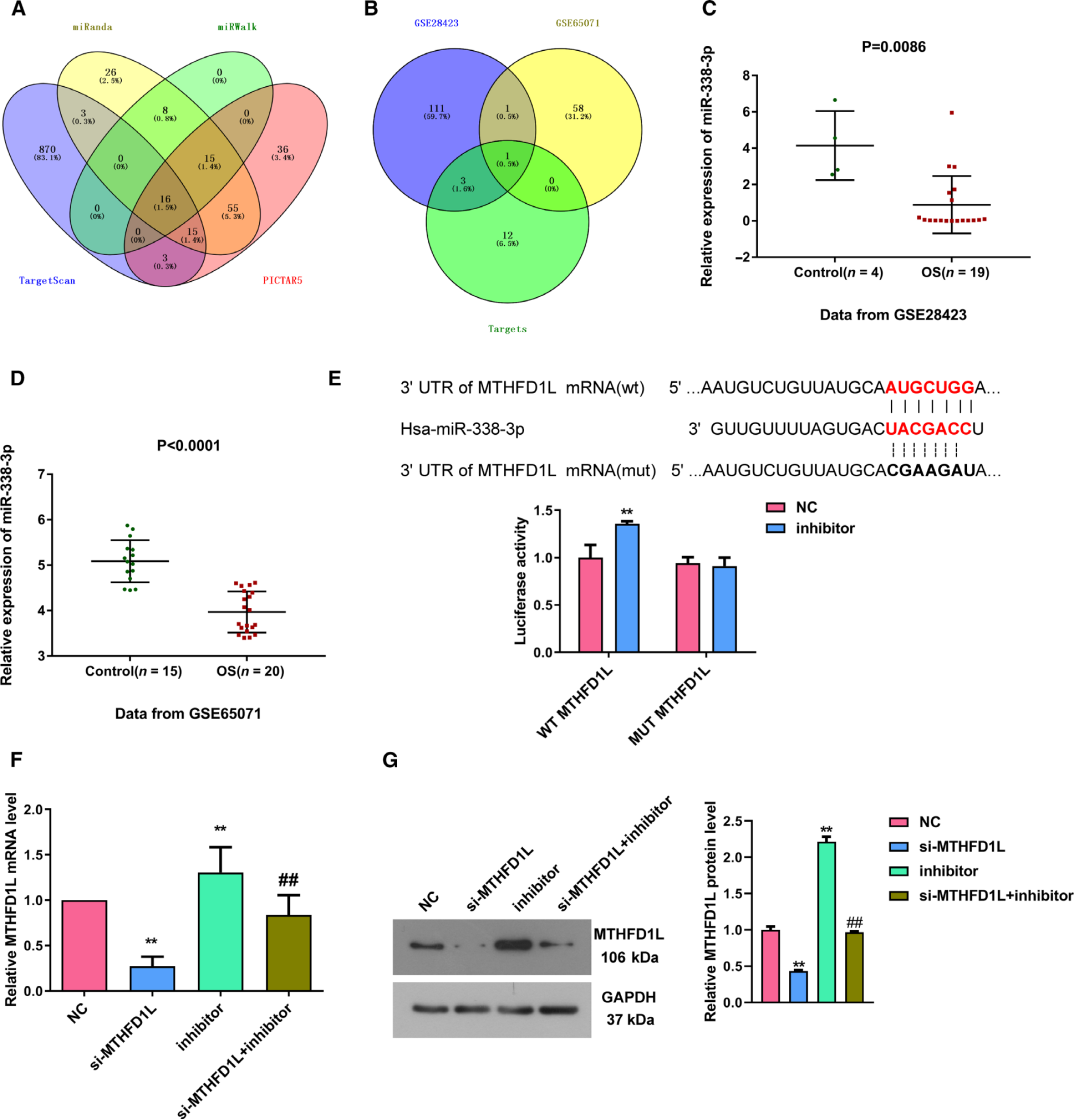
*MTHFD1L* is a direct target of *miR‐338‐3p* in OS cells. (A) The upstream miRNAs of *MTHFD1L* predicted by TargetScan, PICTAR5, miRanda and miRWalk. (B) Intersection among predicted upstream miRNAs of *MTHFD1L*, down‐regulated miRNAs identified by GEO: GSE28423 and GSE65071. (C, D) Expression of *miR‐338‐3p* was markedly decreased in OS tissues compared with controls. (E) Sequences of the binding sites between *miR‐338‐3p* and *MTHFD1L* are shown, and luciferase reporter gene analysis was conducted to determine the relationship between miR‐338‐3p and *MTHFD1L*. (F, G) After MG‐63 and SW1353 cells were transfected with si‐MTHFD1L, *miR‐338‐3p* inhibitor and si‐MTHFD1L + *miR‐338‐3p* inhibitor, the mRNA and protein levels of MTHFD1L were measured by quantitative real‐time PCR and western blotting, respectively. Data are shown as the means ± SD of three independent experiments. The data were statistically analyzed by Student’s *t*‐test or Dunn’s test. **P < 0.01 versus NC group; ^##^ *P* < 0.01 versus si‐MTHFD1L or *miR‐338‐3p* inhibitor group.

miRNAs primarily perform their biological functions via regulating the target genes [19]. As searched via TargetScan, PICTAR5, miRanda and miRWalk, we obtained 16 miRNAs targeting *MTHFD1L* in overlapping part (Fig. 3A). Then, the two datasets GEO: GSE28423 and GSE65071 were used to identify the down‐regulated miRNAs in OS. After intersecting with down‐regulated miRNAs and potential miRNAs that target *MTHFD1L*, only one common miRNA, *miR‐338‐3p*, was left (Fig. 3B). *miR‐338‐3p* was significantly reduced in OS tissues relative to normal controls (Fig. 3C,D). Fig. 3E exhibited the binding sites of *miR‐338‐3p* and *MTHFD1L*. Luciferase activity analysis was subsequently conducted to verify the prediction, and results revealed that the luciferase activity was obviously increased in cells transfected with wt‐*MTHFD1L* and *miR‐338‐3p* inhibitor. There were no changes in the mut‐*MTHFD1L* group after the transfection of *miR‐338‐3p* inhibitor. Furthermore, *miR‐338‐3p* inhibitor strikingly increased the expression of MTHFD1L at both mRNA and protein levels (Fig. 3F,G). The cotransfection of si‐MTHFD1L and *miR‐338‐3p* inhibitor restored the MTHFD1L expression that was decreased or increased by si‐MTHFD1L or *miR‐338‐3p* inhibitor. Herein, these observations supported that *MTHFD1L* is a direct target of *miR‐338‐3p* in OS.

### Arbutin mediates the regulatory action of the *miR‐338‐3p*/MTHFD1L axis in OS cells

**Fig 4:**
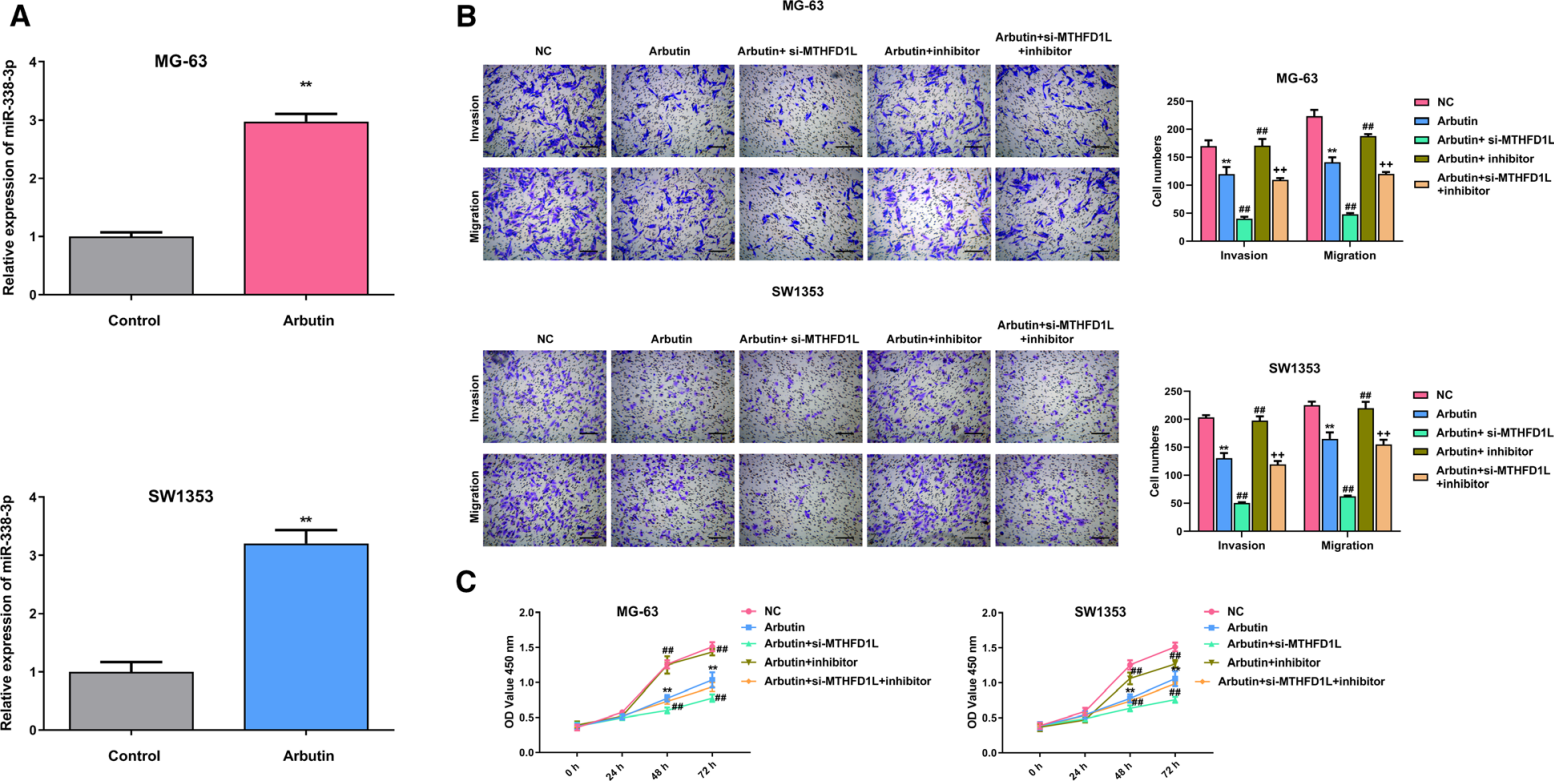
Arbutin mediates the regulatory action of *miR‐338‐3p*/MTHFD1L in OS cells. (A) The expression of *miR‐338‐3p* in Arbutin‐treated OS cells. (B) Transwell migration and invasion experiments were implemented in Arbutin‐treated MG‐63 and SW1353 cells after the transfection of si‐MTHFD1L, *miR‐338‐3p* inhibitor and si‐MTHFD1L + *miR‐338‐3p*. (C) CCK‐8 assay was conducted in Arbutin‐stimulated MG‐63 and SW1353 cells transfected with si‐MTHFD1L or *miR‐338‐3p* inhibitor. Scale bars, 200 μm. Data are shown as the means ± SD of three independent experiments. The data were statistically analyzed by Student’s *t*‐test or Dunn’s test. **P < 0.01 versus control or NC group; ^##^ *P* < 0.01 versus Arbutin group; ^++^ *P* < 0.01 versus si‐MTHFD1L or *miR‐338‐3p* inhibitor group.

After determining the targeted correlation between *miR‐338‐3p* and *MTHFD1L*, we next examined whether *miR‐338‐3p* is implicated in the regulation of MTHFD1L on Arbutin‐stimulated OS cell behaviors. First, we detected the expression of *miR‐338‐3p* in Arbutin‐treated MG‐63 and SW1353 cells. Results manifested that *miR‐338‐3p* was significantly promoted in OS cells as a result of the treatment of Arbutin (Fig. 4A). Compared with the NC group, Arbutin treatment remarkably inhibited OS cells’ invasive and migratory motilities (Fig. 4B). The MTHFD1L silencing further repressed the invasion and migration of Arbutin‐treated OS cells, and this effect was restored by the addition of *miR‐338‐3p* inhibitor. As expected, the transfection of *miR‐338‐3p* inhibitor significantly elevated the invasion and migration capabilities of OS cells after Arbutin stimulation, which was reversed by MTHFD1L knockdown (Fig. 4B). Similarly, the dramatic repression of Arbutin to cell viability was also observed in OS cells (Fig. 4C). More than that, the repressive effect of Arbutin on cell proliferation was enhanced because of MTHFD1L deficiency but abrogated by the transfection of *miR‐338‐3p* inhibitor (Fig. 4C). Collectively, results demonstrated that Arbutin modulates the regulatory action of the *miR‐338‐3p*/MTHFD1L pair in OS cells.

### Arbutin is involved in the AKT/mTOR pathway modulated by the *miR‐338‐3p*/MTHFD1L axis

**Fig 5:**
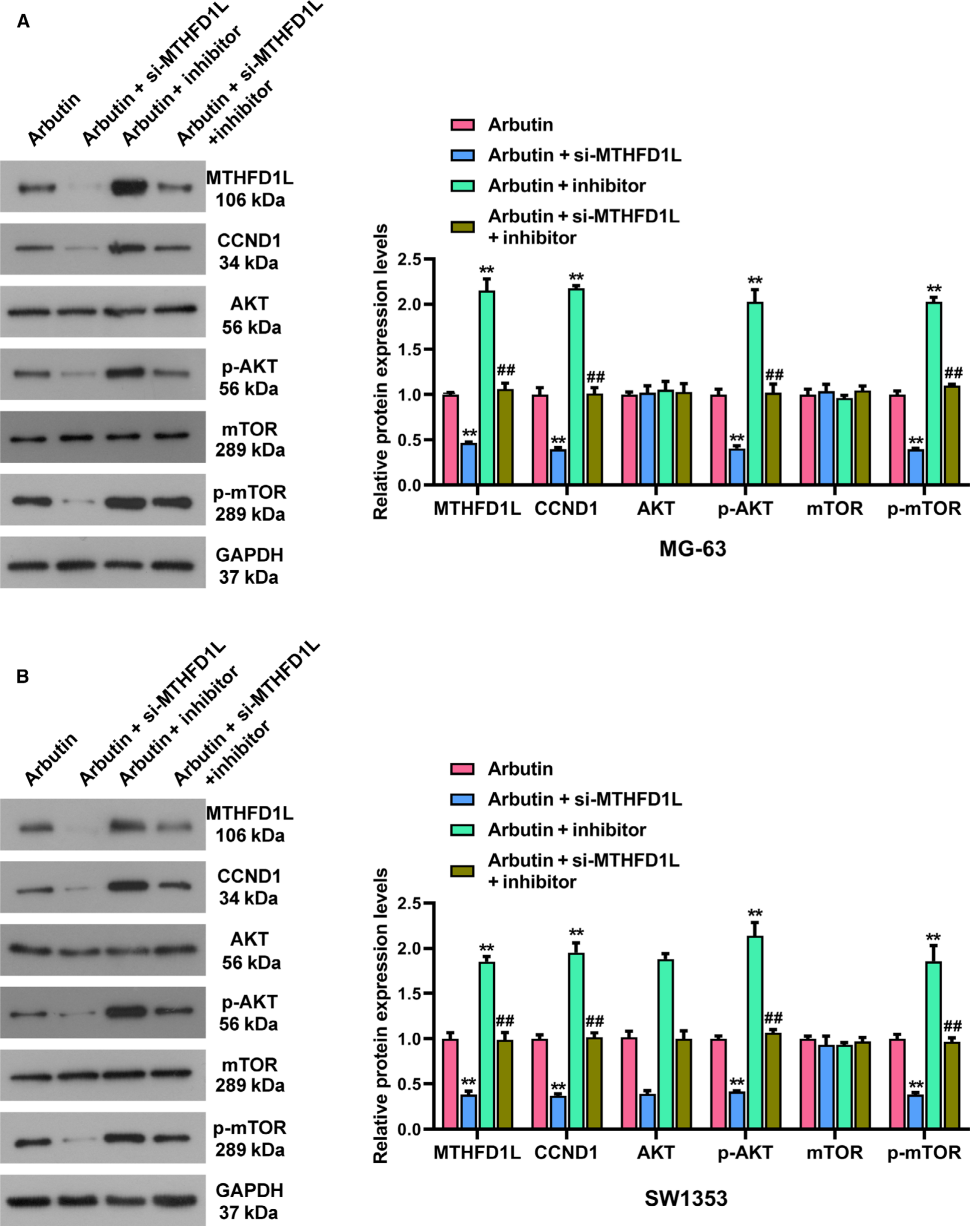
Arbutin participates in the *miR‐338‐3p*/MTHFD1L axis mediating the AKT/mTOR pathway. (A, B) Expressions of MTHFD1L, CCND1, AKT/p‐AKT and mTOR/p‐mTOR were measured using western blot in MG‐63 and SW1353 cells after Arbutin treatment and different transfections. Data are shown as the means ± SD of three independent experiments. The data were statistically analyzed by Dunn’s test. **P < 0.01 versus Arbutin group; ^##^ *P* < 0.01 versus si‐MTHFD1L or *miR‐338‐3p* inhibitor group.

Given the suppressive effect of Arbutin treatment on MTHFD1L expression, we then conducted western blot to estimate whether Arbutin affects the management of the *miR‐338‐3p*/MTHFD1L axis in the AKT/mTOR pathway. In Arbutin‐treated MG‐63 cells, the transfection of *miR‐338‐3p* inhibitor significantly increased MTHFD1L expression, and these conditions were all reversed by the cotransfection of si‐MTHFD1L and *miR‐338‐3p* inhibitor (Fig. 5A). Down‐regulation of *miR‐338‐3p* also elevated the expression of CCND1, and this tendency was overturned by the involvement of si‐MTHFD1L (Fig. 5A). Moreover, the repression of Arbutin treatment on p‐AKT/p‐mTOR was enhanced by silencing MTHFD1L but overturned by *miR‐338‐3p* inhibitor. The individual effects of si‐MTHFD1L and *miR‐338‐3p* inhibitor on AKT/mTOR pathway‐related proteins expressions were abolished by the cotransfection of si‐MTHFD1L and *miR‐338‐3p* inhibitor (Fig. 5A). As expected, the similar phenomena were also observed in Arbutin‐stimulated SW1353 cells (Fig. 5B). All these data suggested that Arbutin might participate in the AKT/mTOR pathway modulated by the *miR‐338‐3p*/MTHFD1L axis.

## Discussion

In this investigation, we harvested evidences supporting the correlation between Arbutin and *miR‐338‐3p*/MTHFD1L in OS. In OS cells, Arbutin treatment significantly inhibited cell viability. Moreover, the expressions of MTHFD1L and AKT/mTOR signaling‐related markers were also decreased by Arbutin treatment. *MTHFD1L* was a potential target gene of *miR‐338‐3p*. Effects of *miR‐338‐3p* and MTHFD1L in OS cells were affected by the stimulation of Arbutin. In summary, these findings determined our hypothesis that Arbutin hindered the development of OS by modulating the effects of *miR‐338‐3p*/MTHFD1L to OS and inactivating AKT/mTOR signaling.

Proliferation and metastasis have been identified as main cosmetic concerns in cancer progression and therapeutics [20]. Arbutin is a naturally occurring hydroquinone, which has been proved to be a cytoprotective agent with insignificant cytotoxicity at high doses [21]. Arbutin plays an inhibitory effect on tumor cell viability, including HCT‐15 human TCC SUP cells and human A375 melanoma cells [10, 22]. Consistent with these prior studies, our data displayed that Arbutin remarkably suppressed cell proliferation of OS cells. Invasion and migration are two major forms for cancer metastasis. Our transwell assay results showed that Arbutin blocked OS cell invasion and migration. Moreover, Arbutin was selected as an inhibitor for MTHFD1L expression. Western blot concluded that the treatment of Arbutin could significantly decrease the MTHFD1L protein expression. Several studies have indicated that Arbutin exerts important roles by targeting miRNAs. Through targeting *miR‐27a*, Arbutin could relieve apoptosis and autophagy of protected high‐glucose‐stimulated HK‐2 cells [23]. The oxidative injury caused by hydrogen peroxide was relieved by Arbutin via regulating *miR‐29a* in retinal ganglion cells [24]. The key mechanism that allows miRNAs to be involved in tumorigenesis is to modulate the expression of targeted genes. Therefore, in the future, we should take measures to focus on the underlying mechanism addressed by Arbutin/miRNA/mRNA in the development of OS.

Bioinformatics analysis in this study manifested that *miR‐338‐3p* was obviously reduced in OS and can directly target *MTHFD1L*, indicating that *miR‐338‐3p* may be an inhibitory regulator for OS development. Existing evidences demonstrated that *miR‐338‐3p* retards ovarian cancer cells proliferation and metastasis via mediating the Wnt/β signaling pathway [25]. *miR‐338‐3p* carries 5‐fluorouracil resistance in *p53* mutant colon cancer cells through regulating the mTOR [26]. *miR‐338‐3p* increased by baicalin exerts an anticancer role in breast cancer [20]. In addition to that, in OS, *miR‐338‐3p* plays the role of a tumor suppressor for OS cell malignant behaviors through inactivating the mitogen‐activated protein kinase pathway [18]. A similar role of *miR‐338‐3p* was found in OS cells by Cao *et al*. [17], who uncovered that *miR‐338‐3p* overexpression markedly repressed OS cells viability, migration and invasion. However, the possible mechanism of *miR‐338‐3p* and putative natural agents have not been clearly explored in OS. Functional *in vitro* experiments displayed that down‐regulation of *miR‐338‐3p* reversed the anticancer role of Arbutin in cell proliferation, migration and invasion. MTHFD1L expression was negatively regulated by *miR‐338‐3p* in OS cells. *MTHFD1L* was a direct target of *miR‐338‐3p*. To further assess the underlying mechanism addressed by Arbutin/*miR‐338‐3p*/MTHFD1L, we conducted *in vitro* assays to measure the impacts of Arbutin/*miR‐338‐3p*/MTHFD1L in cell viability, invasion and migration in OS cells. All data illuminated that *miR‐338‐3p* inhibited cell malignant behaviors in Arbutin‐treated OS cells through directly targeting MTHFD1L.

Abnormal activity of AKT/mTOR signaling is frequent in solid tumors [27]. Its activation significantly facilitates the development of various cancers, including OS [28]. Notably, the prooncogenic role of the AKT/mTOR signaling pathway in carcinogenesis has become a hotspot for the development of drug [29, 30]. Moreover, because our previous study has demonstrated that MTHFD1L expression was enriched in the AKT/mTOR pathway, and we found that silencing MTHFD1L could hinder OS cell aggressiveness by inactivating the AKT/mTOR pathway [12], thereby we implemented a western blot test to expound the correlation between Arbutin/*miR‐338‐3p*/MTHFD1L and AKT/mTOR signaling. AKT/p‐AKT and mTOR/p‐mTOR were crucial markers in the AKT/mTOR signaling pathway [31]. Data observed by us showed that Arbutin induced a decreased level of p‐AKT and p‐mTOR, and knockdown of MTHFD1L strengthened the inhibitory effect of Arbutin on p‐AKT and p‐mTOR expression, whereas the *miR‐338‐3p* inhibitor reversed it. CCND1 functions as a crucial regulator in G1‐to‐S phase and is increased in most human tumors [32, 33]. Its overexpression was related with unfavorable prognosis and tumor metastasis, and it also was involved in the regulatory action of MTHFD1L to OS [34]. The expressional pattern of CCND1 was consistent with p‐AKT and p‐mTOR in Arbutin‐induced OS cells. These observations demonstrated that Arbutin inhibited OS cell aggressiveness via *miR‐338‐3p*/MTHFD1L signaling.

Taken together, to the best of our knowledge, our work was the first study to unearth the involvement of Arbutin on the effects of the *miR‐338‐3p*/MTHFD1L axis to OS development. We concluded that Arbutin exerted antiproliferative and anti‐invasive properties in OS cells through modulating the *miR‐338‐3p*/MTHFD1L axis to inactivate the AKT/mTOR pathway. Nevertheless, how Arbutin modulates the *miR‐338‐3p*/MTHFD1L axis in OS cells has yet to be explored. Thus, our future work would emphasize this mechanism.

## Conflict of interest

The authors declare no conflict of interest.

## Author contributions

C‐QW and LW initiated and designed the study. C‐QW, X‐MW and B‐LL prepared experiments and collected data. C‐QW and Y‐MZ analyzed data. All authors helped to prepare and revise the manuscript.

## Data Availability

The datasets supporting the conclusions of this work are included within the article.
